# Deep Infiltrating Endometriosis in Adolescence: Early Diagnosis and Possible Prevention of Disease Progression

**DOI:** 10.3390/jcm13020550

**Published:** 2024-01-18

**Authors:** Francesco Giuseppe Martire, Matteo Giorgi, Claudia D’Abate, Irene Colombi, Alessandro Ginetti, Alberto Cannoni, Francesco Fedele, Caterina Exacoustos, Gabriele Centini, Errico Zupi, Lucia Lazzeri

**Affiliations:** 1 Gynecological Unit, Department of Surgical Sciences, University of Rome “Tor Vergata”, 00133 Rome, Italy; francesco.martire@ao-siena.toscana.it (F.G.M.); caterinaexacoustos@tiscali.it (C.E.); 2 Department of Molecular and Developmental Medicine, Obstetrics and Gynecological Clinic, University of Siena, 53100 Siena, Italy; giorgi13@student.unisi.it (M.G.); claudiadabate94@gmail.com (C.D.); colombi.irene1@gmail.com (I.C.); ginettialessandro14@gmail.com (A.G.); albertoacannoni@gmail.com (A.C.); gabriele.centini@unisi.it (G.C.); lucia.lazzeri@unisi.it (L.L.); 3 Department of Obstetrics and Gynecology, Fondazione “Policlinico-Mangiagalli-Regina Elena” University of Milan, 20122 Milan, Italy; francesco.fedele@unimi.it

**Keywords:** adolescence, deep infiltrating endometriosis, diagnosis, pelvic pain, treatment

## Abstract

Endometriosis has a prevalence of 10% worldwide in premenopausal women. Probably, endometriosis begins early in the life of young girls, and it is commonly diagnosed later in life. The prevalence of deep infiltrating endometriosis (DIE) in adolescence is currently unknown due to diagnostic limits and underestimation of clinical symptoms. Dysmenorrhea is a common symptom in adolescents affected by DIE, often accompanied by dyspareunia and chronic acyclic pelvic pain. Ultrasonography—either performed transabdominal, transvaginal or transrectal—should be considered the first-line imaging technique despite the potential for missed diagnosis due to early-stage disease. Magnetic resonance imaging should be preferred in the case of virgo patients or when ultrasonographic exam is not accepted. Diagnostic laparoscopy is deemed acceptable in the case of suspected DIE not responding to conventional hormonal therapy. An early medical and/or surgical treatment may reduce disease progression with an immediate improvement in quality of life and fertility, but at the same time, painful symptoms may persist or even recur due to the surgery itself. The aim of this narrative review is to report the prevalence of DIE in adolescents, describe the pathogenetic theories and discuss the management in adolescent women, including the challenging road to diagnosis and the treatment alternatives.

## 1. Introduction

Endometriosis is an estrogen-dependent disease characterized by the presence of endometrial glands and stroma outside the uterine cavity. The prevalence is 10% in premenopausal women. Probably, endometriosis starts early in the life of young girls but is commonly diagnosed later in life [1].

Deep infiltrating endometriosis (DIE) is the most aggressive type of endometriosis, with deep infiltration of tissues leading to subverted anatomy and functionality of vital organs and reduced quality of life [2]. Its prevalence in adolescent age is currently unknown due to the underestimation of clinical symptoms and unsolved diagnostic challenges.

Among clinical symptoms, dysmenorrhea is widely experienced in adolescents affected by endometriosis [3]. Dysmenorrhea may be primary or secondary, and the distinction between the two forms is essential for clinicians.

Endometriosis is the leading cause of secondary dysmenorrhea in adolescents and may be associated with other typical symptoms, including chronic pelvic pain (CPP), dyspareunia, heavy menstrual bleeding and infertility [4]. According to the ESHRE guidelines [5], manifestations suggestive of endometriosis include early menarche, severe dysmenorrhea, dyspareunia, abnormal uterine bleeding (heavy or irregular bleeding), mid-cycle or acyclic pain, resistance to empiric medical treatment (such as painkillers and hormonal therapy) and gastrointestinal and genitourinary symptoms. Furthermore, the disease may present atypically with gastrointestinal symptoms, such as constipation, diarrhea, nausea, vomiting and, less frequently, urinary symptoms [6]. Along with all these symptoms, endometriosis should be suspected in case of frequent absenteeism from school or work during menstruation and early prescription of estroprogestin contraceptives before 18 years of age due to severe dysmenorrhea [7].

Despite the presence of severe painful symptoms, the diagnosis of endometriosis in young patients is challenging. Most often, adolescents are affected by early-stage endometriosis. In these cases, physical examination or instrumental diagnostic imaging may fail to detect the small-sized endometriotic foci [8]. Hence, endometriosis is more commonly diagnosed late after the age of 25, seven to nine years after symptoms’ onset [9].

Although some authors affirm DIE lesions do not grow after the age of 25 [10], some others suggest that endometriosis could be a progressive disease with early onset of DIE [11,12,13,14]. Regardless of who is right, early diagnosis and early treatment may prevent or at least slow down disease progression and have a good impact on both quality of life and fertility. Indeed, patients with advanced stages of disease who undergo surgery have an increased risk of intra- and post-operative complications [15] and may show symptoms persistence due to surgery itself, particularly in the presence of posterior DIE and, less frequently, in the case of anterior DIE [16,17].

Defining the path toward an early diagnosis of endometriosis is of utmost importance and may change the approach to the disease.

The aim of this narrative review is to report the prevalence of DIE in adolescents, describe the pathogenetic theories and discuss the management in adolescent women, including the challenging road to diagnosis and the treatment alternatives.

## 2. Search Methodology

We conducted an electronic literature search using the MEDLINE database (accessed via PubMed) to identify all English language articles on DIE and adolescence from inception to October 2023. Combinations of the following keywords and Medical Subject Headings (MeSH) search terms were used to screen and identify studies: “Adenomyosis” (unique ID: D062788), “Adolescent” (unique ID: D000293), deep infiltrating endometriosis, “Diagnosis” (unique ID: D003933), “Diagnostic Imaging” (unique ID: D003952), “Early diagnosis” (unique ID: D042241), “Endometriosis” (unique ID: D004715), “Magnetic Resonance Imaging” (unique ID: D008279), symptom, “Therapy” (unique ID: D013812), “Ultrasonography” (unique ID: D014463), ultrasound.

Original articles (randomized and non-randomized clinical trials, prospective observational studies, retrospective cohort studies and case–control studies) and review articles were considered available for the purpose of this review. The reference lists of each eligible study were also searched for additional articles.

Articles were considered eligible when they met the aim of this narrative review, i.e., providing an overview of DIE in adolescence (patients aged 12–20 years) with a particular focus on early diagnosis and early treatment. We excluded data from the hybrid study’s population, i.e., adolescents and young women combined and impossibility to extract data on the adolescent group.

The research was performed independently by two authors (F.G.M. and L.L.) who thoroughly read articles meeting the inclusion criteria.

One hundred and thirty-seven articles were included to highlight all the clinical aspects of the topic, starting from the pathogenetic theories, going through the prevalence of the disease and imaging diagnosis and ending with the potential treatment options.

## 3. Pathogenesis of DIE in Adolescents

The pathogenesis of DIE is unclear, with several hypotheses, theories and even speculations [18]. The absence of animal models mimicking human endometrium functions and simulating the potential specific growth mechanisms of DIE feeds the debate. There is even more uncertainty on the pathogenesis of DIE in adolescents. It is probably caused by the same combination of factors postulated for adults, with some slight differences that promote adolescent DIE [19].

Neonatal menstruation may explain some forms of endometriosis in adolescence [20,21]. In most girls, the functional transition to fully responsive endometrial cells is achieved during adolescence. In about 5% of neonates, newborns’ endometrium can be sensitive to pregnancy’s high maternal progesterone levels, and menstrual shedding can occur early in the first week of life. In these neonates, retrograde menstruation occurs because of the relatively long cervix and thick cervical secretions [13]. Retrograde endometrial cells contain stem cells along with perivascular mesenchymal stem/stromal cells together with niche cells, which implant in the peritoneal mesothelium, leading to a very early and inactive stage of disease. Obviously, the risk of retrograde menstruation can be increased by obstructive Müllerian anomalies [22].

Indeed, some prenatal factors may also be associated with the onset of DIE in adolescence, such as preeclampsia and low birth weight [21]. The underlying mechanism would be the fetal hypoxic environment caused by placental insufficiency that would increase endometrial sensitivity to progesterone [23] and the altered platelet activation leading to augmented angiogenesis [13]. It is hypothesized that the presence of a fetal hormonal milieu then facilitates the development of DIE [24].

However, endometriosis in prepubertal girls is very rare, and neonatal menstruation [20] alone cannot explain the progression from a “very early-stage” disease (i.e., endometrial attachment to the mesothelium) to DIE disease [25]. The quiescent and early-implanted endometrial cells are probably reactivated by increasing ovarian activity during menarche and thelarche with the growth of endometriosis lesions [18,20,26]. Surrounding smooth muscle hyperplasia may play a role in this process by supporting endometrial glands and stroma to dive deep into fibromuscular tissues [25,27]. The implantation would be encouraged by macromolecular oxidative damage and recurrent tissue injury and repair, with destruction of the peritoneal mesothelium and chronic inflammation due to peritoneal iron excess and local bleeding of ectopic endometriotic lesions [18,25].

Nevertheless, the progressive evolution of the disease is debated and, to date, not yet clearly defined [25].

The metaplasia theory was proposed more than 50 years ago to explain endometriosis development in women without a uterus [28]. This theory stems from the old histological concept that the histological aspect of a mature cell can transform into that of another mature cell. Metaplasia is considered a consequence of stem cells’ growth activity. Regarding the pathogenesis of endometriosis, stem cells of endometrial [29,30] or peritoneal [12,31,32] origin have been described, probably linked to genetic changes [18]. The existence of specific endometrial cells arising from reiterated ultra-microtrauma of the junctional zone called “pale cell” [33,34] was hypothesized to be the origin of endometriosis and adenomyosis.

Koninckx et al. presented the endometriotic disease theory in 1994 [35]. The term “endometriosis” was used to describe asymptomatic lesions, and “endometriotic disease” referred to several different lesions (deep, cystic or typical) leading to clinical manifestation of the diseases. According to this theory, some cellular or genomic incidents may cause the transition from “endometriosis” to “endometriotic disease”. Typical cystic and deep endometriosis lesions would consist of abnormal cells developing outside of the uterine environment and arising from the endometrium or other organs, such as bone marrow, embryonic cells or stem cells.

The immunological theory has been proposed to explain the migration of endometriotic cells outside the uterus and their survival in the ectopic environment [36,37,38]. The existence of this pathogenetic mechanism is supported by the high prevalence of autoimmune diseases coexisting with endometriosis, such as systemic lupus erythematosus, rheumatoid arthritis, Sjogren’s syndrome, hypothyroidism and fibromyalgia.

Evasion of immune surveillance and numerous cytokines implicated in angiogenesis and the activation of proinflammatory pathways play a key role in this process [39]. Elevated levels of circulating and local cyclooxygenase-2, tumor necrosis factor-α, prostaglandin-E2, growth factors, cytokines and angiogenic factors are implicated in proinflammatory signaling [40]. Increased interleukin-1α, interleukin-6 and interleukin-8 promote cell growth and angiogenesis, and fibronectin allows attachment of ectopic cells. The proliferation of fibroblasts and endometrial cells is aided by elevated levels of vascular endothelial growth factor, macrophage-derived growth factor and monocyte chemotactic protein [40].

In addition, some studies have shown changes in complement proteins in patients with endometriosis. According to the recent literature, complement proteins, and especially the C6 fraction, would appear to be implicated in the early stages of the disease [41].

Genetic and epigenetic changes have been studied in the pathogenesis of endometriosis recently [42]. About 50% of endometriosis lesions are linked to hereditary factors [43,44]. The similar prevalence and age of symptoms’ onset in twin sisters seems to confirm the major role of hereditary genetic factors [45,46,47]. Researchers have identified some genes that may be involved in the pathogenesis of the disease. Two aberrant loci (i.e., 10q26 and 7p13–15) coding for harboring genes, including CYP2C19, INHBA, SFRP4 and HOXA10, had a positive correlation with endometriosis [48]. However, probability logarithm results were too low to identify a major responsible gene. Genome-wide scanning resulted in ten significant loci, six of which had a strong association with the development of endometriosis [49,50]. Recently, Rahmioglu et al. published a genome-wide association meta-analysis with over 60 thousand cases and 700 thousand controls [51]. They identified 42 genome-wide significant loci comprising 49 distinct association signals able to explain up to 5.01% of disease variance, mostly ovarian endometriosis. Some of them (i.e., SRP14/BMF, GDAP1, MLLT10, BSN and NGF) were associated with pain perception/maintenance.

One mechanism potentially involved in the genetic pathogenesis of endometriosis is the loss of heterozygosity, also called “first–second hit hypothesis” [18]. The occurrence of a second genetic hit in a patient already carrying a first hit may explain both the development and the inheritance of the disease.

Currently, there is a lack of certain and reproduced clinical data about the genetics of endometriosis, and further research will help clinicians in the early identification of the disease and earlier targeted therapies for symptoms.

In the past decades, epigenetic changes have been investigated to assess their role in favoring endometriosis growth during fetal life [52,53,54]. Some studies observed changes in methylation and demethylation of DNA in endometriosis tissue by comparing it with similar changes in the normal endometrium [55,56,57]. Some others unveiled the detrimental action of dioxin [58,59] and total body radiation [60,61], as they were both correlated to endometriosis development by giving epigenetic or genomic effects [62]. Many aberrations in the DNA of endometriosis patients have been described, but so far, they fail to explain the whole genetic pathway of endometriosis.

In conclusion, the pathogenesis of deep endometriosis and its early onset in adolescence remain controversial. We can identify two literature factions that are reassessing the historical mechanism of endometriosis development and adapting them to adolescents: on the one hand, there is the belief that a normal cell goes through local or immunologic factors, which induce its growth, transformation or metaplasia; on the other hand, deep endometriosis may be the consequence of a genetically or epigenetically modified cell.

## 4. Prevalence of DIE in Adolescents

Despite the efforts from researchers, the real prevalence of endometriosis in adolescence is, so far, an unsolved enigma. The estimates vary widely, with ranges between 19% and 100% [63,64], reflecting the uncertainty about this aspect of adolescent endometriosis.

The estimated prevalence of adolescent DIE is even more uncertain. DIE prevalence changes according to the inclusion criteria of each study’s population (i.e., overall vs. symptomatic adolescents) and the diagnostic employed, which includes imaging, with detection of ultrasonographic or magnetic resonance imaging (MRI) features suggestive of DIE, and surgery, with direct visualization of the disease [65]. Collection of medical history, especially regarding painful symptoms associated with DIE, is not diagnostic but may drive towards an early diagnosis [66,67,68,69].

Martire et al. have assessed the ultrasonographic presence of different forms of endometriosis in adolescent girls, including ovarian, DIE and adenomyosis [70]. The study population included 270 patients aged 12–20 years who received pelvic ultrasonography (US) examination (transvaginal or transrectal) for different clinical indications. Among them, endometriosis was detected in 13.3% of the cases. DIE affected 3.7% of the whole study population, whereas OMA was the most frequently involved localization, standing at 8.1%. USL was the most common site of DIE, with seven cases (2.6%). Moreover, they evaluated the correlation between ultrasound findings of DIE and symptoms. Affected adolescents were heavily impacted by painful symptoms, such as dysmenorrhea (100%), dyspareunia (60%), dysuria and dyschezia (20% each). Seventy-one percent of patients with USL involvement suffered from dyspareunia. However, these data should be interpreted with caution considering the limited DIE casuistic of the study (10 patients).

The same research group later published a retrospective observational study that showed sonographic signs of posterior DIE in 10.6% of adolescent patients who had undergone ultrasonographic exams for severe dysmenorrhea [71]. Instead, no signs of anterior DIE were detected.

Regarding DIE prevalence on MRI diagnostic, Millischer et al. prospectively investigated the prevalence of DIE and OMA in a series comprising 308 adolescents aged 12–20 years who underwent MRI for severe dysmenorrhea [72]. In this cohort, MRI was positive for DIE and/or OMA in 39.3% of the cases. In particular, DIE was observed in 88.4% of the cases, with predominantly retrocervical involvement (87.6%) and coexisting OMA in 9.1% of the cases. This study also highlighted the increasing prevalence of overall endometriosis, rising from 16.7% at the age of 12–13 years to 60.5% at 20 years, and revealed no site-specific distribution along with age variations.

In one of the largest series in the literature with surgical diagnoses of endometriosis (population study: 63 patients), Yang et al. reported 44.4% had a diagnosis of DIE divided into 28.6% involving the rectovaginal pouch and 31.8% involving the uterosacral ligaments [73]. Moreover, DIE affected women without genital tract malformations more frequently (54.2% vs. 13.3%, *p* = 0.005).

More recently, Stochino-Loi et al. reported a 31.2% prevalence of deep endometriosis nodules in a series of 32 patients who underwent surgery for endometriosis [74]. Most of them had unilateral uterosacral localization (15.6%), but some of them also had rectosigmoid disease (6.2% for rectal and sigmoid nodules each).

In a systemic review published in 2013, Janssen et al. showed an overall prevalence of visually confirmed endometriosis was 62% in all adolescent girls undergoing laparoscopic investigation for chronic pelvic pain [75]. This percentage rose to 75% and 70% in girls with chronic pelvic pain resistant to treatment and in girls with dysmenorrhea, respectively. According to the review, the overall prevalence of moderate–severe endometriosis, classified by the American Society of Reproductive Medicine, was 32%.

## 5. DIE Symptoms

According to the European Society of Human Reproduction and Embryology (ESHRE) guidelines [5], offering surgery to young women for a histological diagnosis of endometriosis is no longer considered appropriate nowadays. Therefore, imaging exams and the collection of medical history assume a central role in the diagnostic workup towards an early diagnosis of endometriosis. Indeed, several studies suggest the use of a combination of a patient’s clinical history and imaging exams to increase the chance of diagnosis [70,71,76].

Regardless of the methodology employed for diagnosis, adolescents presenting with symptoms suggestive of endometriosis, and hence DIE, should be referred to referral centers to avoid diagnostic delay [76].

Most patients with endometriosis refer dysmenorrhea in adolescence or at a young age [77,78]. According to Di Vasta et al., dysmenorrhea can be the presenting symptom of adolescent endometriosis in over 90% of them [66]. It starts at menarche in half of adolescent patients, is frequently associated with nausea (69.5%) with no vomiting (75%) and is described as severe in 63% of the cases [66]. The distinction between primary (menstrual pain with an organic disease) and secondary dysmenorrhea (menstrual pain without an organic disease) should be mandatory when addressing adolescent patients. Indeed, endometriosis represents the main cause of secondary dysmenorrhea in adolescents [76,78].

In the literature, dysmenorrhea has been inconsistently linked to geographical factors [79,80], whereas the presence of DIE can be caused by environmental factors [52,53,54,55,56,57,58]. However, no study has ever assessed a concrete relationship between dysmenorrhea, DIE, the environment and geographical factors in adolescents.

Dysmenorrhea is often neglected, and it is viewed as temporary and natural [81], despite having a high impact on women’s lives [82]. Dysmenorrhea should always be investigated, considering the prevalence of endometriosis in adolescents and young women who refer this symptom, especially when it is severe [71,72] and tends to remain stable over time [68]. Education programs have been delivered in some nations with the aim of making adolescent patients aware of the symptomatology associated with endometriosis [69], enhancing early diagnosis. There is also increasing awareness of the power and amplitude that social media and e-learning may provide in the education of both patients and caregivers [83]. For example, in 2024, the Erasmusplus project entitled ‘Mona Lisa smile’ (Project n. 2022-1-IT01-KA220-VET-000087262) is expected to improve health professionals’ knowledge of the disease and provide them with an easily available tool to identify adolescents at risk through the development of an e-learning platform and an electronic device software (i.e., mobile phone app) [84].

Dysmenorrhea may also be important due to its correlation with specific disease locations, being more frequently associated with posterior DIE and adenomyosis rather than ovarian endometrioma [85]. However, this association has not been investigated in adolescent patients, and further studies are needed to assess the relationship between the DIE site and the specificity of symptoms [38].

Other suggestive symptoms, such as dyspareunia, chronic pelvic pain (CPP) or abnormal uterine bleeding (AUB), may accompany dysmenorrhea, and the coexistence of these symptoms increases the possibility that endometriosis is present [71,76,86]. A combination of dysmenorrhea, chronic pelvic pain and dyspareunia can be the presenting triad of adolescent endometriosis in 58% of the cases, as reported by Pino et al. [86].

Chronic acyclic pelvic pain is more likely to occur in adolescents rather than adults [87,88,89,90]. It is strictly related to endometriosis when it is severe and drug-resistant [75,89] but luckily seems to have a decrease in intensity over the years [68] despite starting with higher intensity in younger patients [90]. Severe chronic acyclic pelvic pain may reflect central sensitization, leading to treatment failure and association with other different painful diseases [68,91,92]. When chronic pelvic pain is reported, many other gynecologic (i.e., fibroids, adenomyosis, pelvic inflammatory disease, ovarian mass, hematocolpo) and non-gynecologic organic disorders should be excluded.

Among pelvic painful symptoms, dyspareunia can be associated with DIE and a more severe disease in adolescents [93]. However, much like infertility, it is difficult to investigate due to sexual behavior and pregnancy desire rates in these types of patients.

Gastrointestinal and genitourinary symptoms in adolescents can be important indicators of endometriosis, particularly suggesting the presence of posterior DIE and adenomyosis [5,71]. Endometriosis can mimic the clinical features of several organic pathologies, including inflammatory bowel diseases, irritable bowel syndrome, Meckel’s diverticulum, celiac disease and sub-acute/chronic appendicitis [94,95,96,97], imposing a multidisciplinary approach to exclude gastrointestinal diseases. Noteworthy, these autoimmune-based inflammatory pathologies can often be associated with endometriosis [98].

## 6. The Role of Imaging in Adolescence DIE Diagnosis

According to the literature, endometriosis in adolescents who underwent surgery is more frequently an early-stage disease, according to the rASRM classification [71,75,89,99]. Additionally, endometriomas are more common in adult women compared to adolescents [88,100]. Therefore, there is a concrete possibility of overlooking stage I/II deep infiltrating endometriosis among adolescents by using imaging diagnostics that may not be able to detect direct signs of the disease.

MRI or US seem to provide similar accuracy in excluding ovarian or deep endometriosis localizations but have not been validated yet in studies investigating an adolescent population [101,102,103]. However, considering that both MRI and US show high accuracy in detecting endometriosis in young patients, the preferred diagnostic approach in adolescents should be ultrasonographic [76,104]. US holds several advantages as compared to MRI in terms of lower costs and wider availability, both for the patients and for the caregivers.

Transvaginal ultrasound (TVS) is preferred in the case of sexually active adolescent patients with suspected endometriosis. However, transrectal sonography (TRS) can be offered in virgin puberal patients, with similar diagnostic accuracy to the TVS technique [70,76,105]. The role of TRS in the evaluation of posterior compartment endometriosis in adults has been investigated both by Ohba [106] and Koga [107]. Both studies showed good diagnostic performances; however, larger studies evaluating adolescent patients are needed to confirm their data. The transabdominal approach is always feasible and can exclude Mullerian obstructive anomalies but plays a minor role due to its intrinsic technical limitations (i.e., the impossibility of performing tenderness-guided investigation) in detecting endometriotic lesions, apart from endometriotic ovarian cysts [108,109]. Notwithstanding the lower invasiveness of TRS and transabdominal sonography exams, care should be taken in any case when approaching adolescent patients due to high levels of anxiety associated with both diagnostic procedures [110].

**Figure 1:**
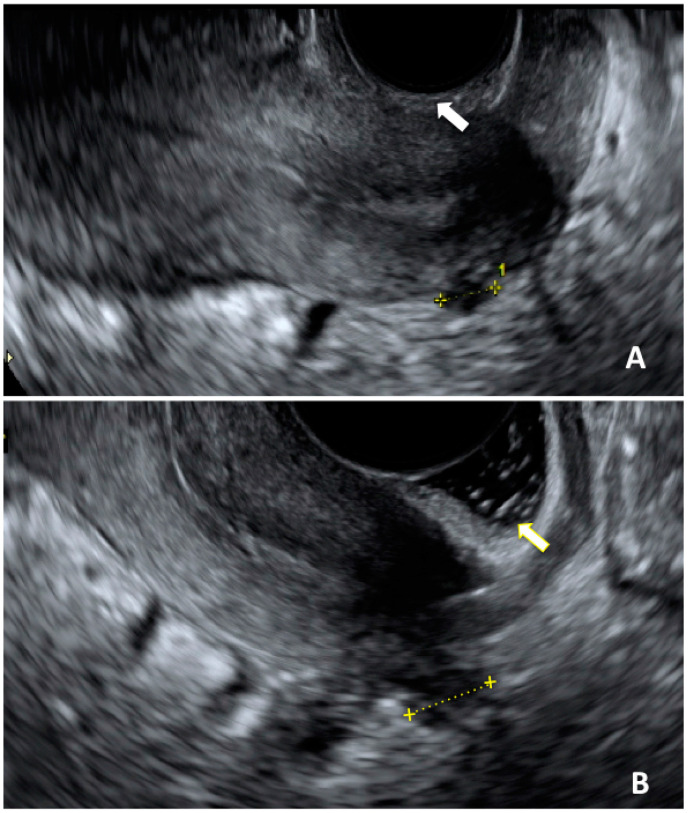
Ultrasonographic image of a DIE nodule affecting uterosacral ligament. (**A**): acquisition of DIE nodule length in a sagittal plane without sonovaginography (white arrow); (**B**): same acquisition performed with sonovaginography (yellow arrow).

Up to now, US accuracy for adolescent deep infiltrating endometriosis has been poorly investigated. The diagnostic performance of the exam compared to a reference “technique”, such as surgery, is difficult to estimate due to the relatively low percentage of patients undergoing surgery for endometriosis [14,71,89]. In a recently published retrospective study, Martire et al. detected isolated DIE in 3.7% of patients, mostly located at the uterosacral ligaments (Figure 1) [70]. The presence of concurrent symptoms was high, especially dysmenorrhea, and this combination enhances the diagnostic performance of US alone [66,71,74,94].

Moreover, there are no data on the reproducibility of US for adolescent endometriosis among expert sonographers and gynecologists with different expertise. The literature advocates for experienced operators in order to avoid or shorten diagnostic delays [111].

Thus, keeping in mind the dark corners of US and its uneven acceptability, further or alternative investigations are advisable in the case of symptomatic adolescents [8,89] with no clear diagnosis or when a US exam is not applicable nor accepted. In these cases, MRI may be a valuable option [112,113,114].

Millischer et al. prospectively assessed the prevalence of endometriosis through MRI exam, showing a high prevalence of isolated DIE (79.6%) and retrocervical area involvement (87.6%). However, no reference technique was considered, much like the mentioned US studies, preventing any conclusions on MRI accuracy for endometriosis detection in adolescents. This study has also been questioned in terms of diagnostic performance for the uterosacral involvement [104]. Indeed, in adults, MRI shows great value for the detection of DIE implants [115], but the detection rate can dramatically decrease to 33% in the case of USL nodules [103], particularly if not performed by expert operators [104].

The added value of MRI is undeniable in the case of DIE implants of the upper rectum or the small bowel, where US is obviously intrinsically limited [103]. Nevertheless, these data should be put into perspective since bowel disease, especially of the upper gastrointestinal tract, is apparently uncommon in adolescent age [71,72,74,75,87,116].

Overall, the role of imaging in the diagnosis of adolescent DIE is unneglectable in terms of low-invasiveness and feasible applicability, but it is probably limited by the common early stage of the disease that makes it difficult to visualize DIE by US or MRI. Common sense would lead to diagnostic laparoscopy as the main diagnostic instrument, but clinicians should always keep in mind the usefulness and potential harm (i.e., wide pelvic dissection) of diagnostic laparoscopy when not driven by adequate preoperative assessment, especially in adolescents with expectations for reproduction while facing chronic disease.

## 7. Adenomyosis and DIE in Adolescence

Adenomyosis is a chronic disease involving the myometrium characterized by the presence of ectopic endometrial and stromal tissue. It is linked with a spectrum of painful symptoms, such as dysmenorrhea, dyspareunia and pelvic pain, and clinical signs, such as heavy menstrual bleeding and infertility [117].

The prevalence of adenomyosis among women is yet to be defined: the data based on histological evaluation of hysterectomy samples report ranges between 15 and 55% [118].

Adenomyosis can affect adolescent patients with varying prevalence ranging from 5% to 17.4% [72,119,120], often in a mild to moderate form with potential clinical implications [120,121]. It is usually diagnosed with noninvasive exams, including ultrasound and MRI [72,120,121]. Adenomyosis may reveal itself in diffuse or focal forms, such as myometrial cysts or adenomyomas [122].

While it may be a rare condition, in the presence of painful pelvic symptoms or heavy menstrual bleeding in an adolescent patient, it should be thoroughly excluded, particularly when coexisting with DIE [120]. Indeed, several previous studies reported a coexistence of adenomyosis and DIE in around 45–50% of cases, mostly associated with posterior compartment disease [70,71,111,121]. Investigation of the presence of adenomyosis in adolescent DIE patients is of utmost importance to achieve appropriate medical and/or surgical management.

**Figure 2:**
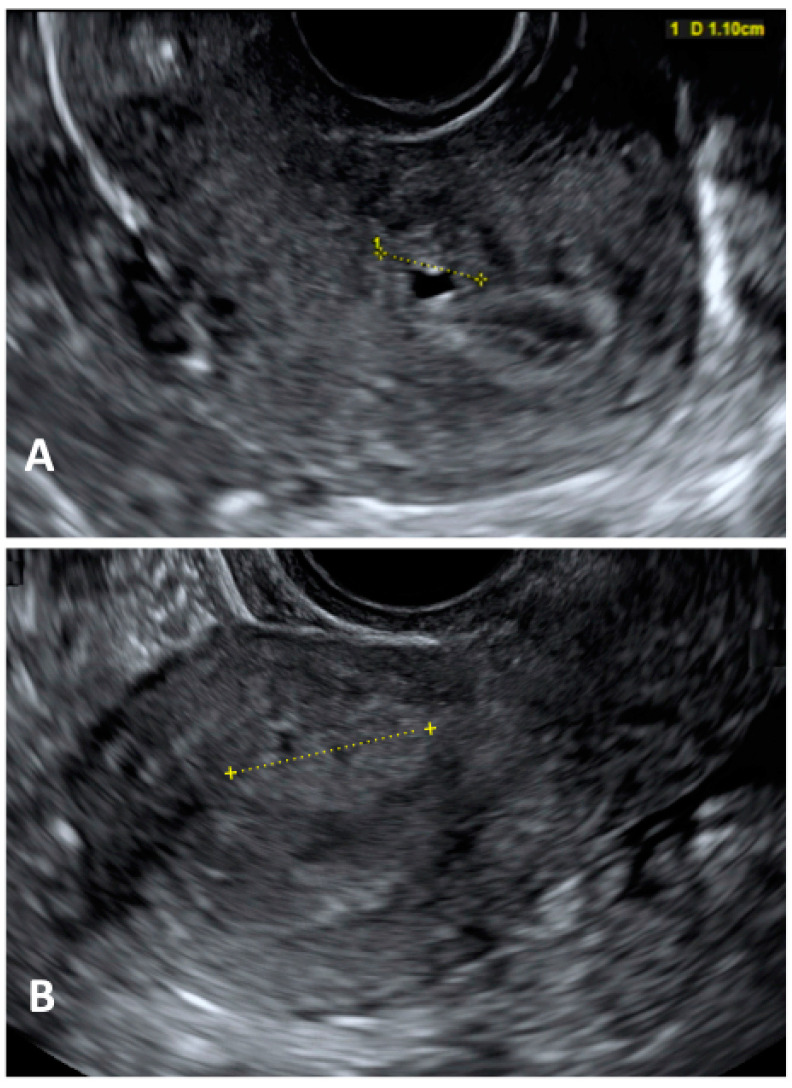
Ultrasonographic image of a focal adenomyosis. (**A**): measurement of a myometrial anechoic cyst; (**B**): measurement of a hyperechoic myometryal island.

Diagnostic laparoscopy should not be employed as the first diagnostic assessment in this group of patients because it may lead to overtreatment and unnecessary invasive procedures (Figure 2). US should be the first-line imaging exam despite the potential overlapping US characteristics with fibroids in patients undergoing hormone therapy [123].

Regarding the treatment of adenomyosis, the main goal is preserving fertility. The therapeutic management, whether pharmacological or surgical, should evaluate the severity of the symptoms, the feasibility of hormone therapy, the very low risk for malignancy irrespective of imaging appearance [124,125] and the possible coexistence of DIE.

## 8. Management

**Figure 3:**
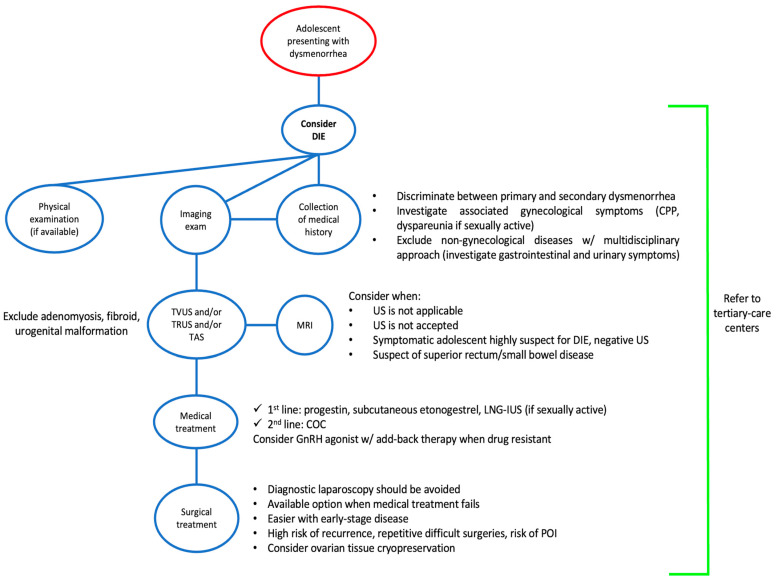
Flowchart for the management of deep infiltrating endometriosis in adolescents. Abbreviations: COC, combined oral contraceptive; CPP, chronic pelvic pain; DIE, deep infiltrating endometriosis; GnRH, gonadotropin releasing hormone; LNG-IUS, levonorgestrel intrauterine system; MRI, magnetic resonance imaging; POI, premature ovarian insufficiency; TAS, transabdominal ultrasound; TRUS, transrectal ultrasound; TVUS, transvaginal ultrasound; US, ultrasound; w/, with.

The treatment of DIE in adolescents should be aimed at reducing painful symptoms, suppressing or controlling the progression of the disease and safeguarding future fertility [126] (Figure 3).

As highlighted in the ESRHE guidelines [5], combined oral contraceptives or progestins are recommended to control the painful symptomatology. Progestin therapy involves the use of Dienogest 2 mg/die and Norethindrone acetate 15 mg/die, both effective and well tolerated in adolescents; alternatively, the subcutaneous implant of etonogestrel, used successfully in adult patients but with limited experience in adolescents, could be proposed; in addition, and only for sexually active patients, LNG-IUS could represent an option [14,63,88].

Progestin therapy should be considered the first-line therapy due to its antiestrogenic effect that should at least slow down DIE growth [63].

However, while being highly effective, the prolonged use of Dienogest as a progestin-only hormonal therapy in puberal patients may be associated with decreased bone mineral density [127].

As second-line treatment, GnRH agonists may be an option [63]. They should be prescribed only in adolescent patients who previously underwent endometriosis surgery and are refractory to other medical therapies (hormonal contraceptives or progestogen therapy). Their mechanism lies in the suppression of estrogen levels, which also brings several side effects. Prescription of an add-back therapy is mandatory, as well as that of vitamin D and calcium, along with appropriate monitoring of bone mineral density [128].

However, there is not a single best medical treatment for endometriosis in adolescents, and therapy must be tailored to each patient.

The optimal timing for surgical treatment in adolescent DIE patients has not yet been established. Since endometriosis could be a progressive disease [129], whether treating endometriosis early in its clinical expression with surgery or employing only hormonal medical therapy to inactivate the foci of disease and reduce painful symptoms [130] remains an open discussion.

As already stated here, many authors consider endometriosis a chronic disease [131], while others believe it to be an eradicable disease [132]. Performing surgery at an early stage might eradicate the initial disease while preventing the difficulties related to the treatment of advanced disease [133], and it has been observed that the complexity of surgical procedures progressively increases from adolescence until the age of 30 years, thus suggesting that the severity of the disease could follow the same trend [74]. On the other hand, surgical treatment early in life is often one of the causes of repetitive surgery, adhesion syndrome and the persistence of painful symptoms due to the surgery itself and should only be performed in cases where is strictly necessary, such as in symptomatic young patients not responding to medical therapy.

Despite the lack of data in the literature regarding fertility preservation in adolescents affected by DIE, ovarian tissue cryopreservation might be considered when the surgical treatment cannot be postponed [134,135]. Most likely, adolescent patients will not seek pregnancy in the short–medium term and may suffer from DIE recurrence and associated repetitive surgeries [134,136]. Oocyte cryopreservation is advocated in young patients affected by ovarian endometriosis, especially when the disease is at an early stage and no previous surgeries have been performed [135]. Therefore, considering the possible coexistence of endometriomas and DIE in adolescent age and the higher risk of premature ovarian insufficiency linked to the early presentation of disease and its consequences, ovarian tissue cryopreservation may be offered to adolescents who undergo surgical treatment upon careful counseling [134].

Surely, according to the ESHRE guidelines [5], offering surgery to provide a histological diagnosis to adolescent patients experiencing chronic pelvic pain is no longer acceptable.

## 9. Conclusions

The diagnosis of DIE in adolescent patients is particularly challenging and can be easily overlooked.

DIE should always be considered in the differential diagnosis of adolescents presenting with painful symptoms, especially secondary dysmenorrhea. Adenomyosis may coexist with DIE; therefore, clinicians should carefully evaluate these patients and choose the most adequate therapeutic approach.

A timely diagnosis is crucial and can be achieved with a combination of medical history, physical examination, ultrasound and/or MRI. Indeed, DIE is more frequently encountered at an early stage; hence, imaging exams alone may fail to detect small lesions. Referral to a tertiary care center is advisable to allow the best management of such a complex, debilitating and chronic disease. Dedicated specialists should provide information on the long-term management and need for follow-up of DIE that starts in adolescence.

Appropriate treatment includes progestins as a first-line option, followed by combined oral contraceptives and GnRH analogues in selected cases. When medical treatments fail to improve the quality of life or reduce the progression of disease, surgery should be employed, and ovarian tissue cryopreservation might be considered.

## Data Availability

Not applicable.
